# Sperm preparedness and adaptation to osmotic and pH stressors relate to functional competence of sperm in *Bos taurus*

**DOI:** 10.1038/s41598-021-01928-6

**Published:** 2021-11-19

**Authors:** Maharajan Lavanya, Santhanahalli Siddalingappa Archana, Divakar Swathi, Laxman Ramya, Arunachalam Arangasamy, Balakrishnan Binsila, Arindam Dhali, Narayanan Krishnaswamy, Sanjay Kumar Singh, Harendra Kumar, Muniandy Sivaram, Sellappan Selvaraju

**Affiliations:** 1 Reproductive Physiology Laboratory, Animal Physiology Division, ICAR-National Institute of Animal Nutrition and Physiology, Adugodi, Bengaluru, 560030 India; 2 Department of Animal Reproduction, Indian Veterinary Research Institute, Izatnagar, Bareilly, 243122 India; 3 Omics Laboratory, Bioenergetics and Environmental Sciences Division, ICAR-National Institute of Animal Nutrition and Physiology, Adugodi, Bengaluru, 560030 India; 4 Indian Veterinary Research Institute, Bangalore, 560024 India; 5 Southern Regional Station, ICAR-National Dairy Research Institute, Adugodi, Bengaluru, 560030 India

**Keywords:** Animal physiology, Mechanisms of disease

## Abstract

The adaptive ability of sperm in the female reproductive tract micromilieu signifies the successful fertilization process. The study aimed to analyze the preparedness of sperm to the prevailing osmotic and pH stressors in the female reproductive tract. Fresh bovine sperm were incubated in 290 (isosmotic-control), 355 (hyperosmotic-uterus and oviduct), and 420 (hyperosmotic-control) mOsm/kg and each with pH of 6.8 (uterus) and 7.4 (oviduct). During incubation, the changes in sperm functional attributes were studied. Sperm kinematics and head area decreased significantly (*p* < 0.05) immediately upon exposure to hyperosmotic stress at both pH. Proportion of sperm capacitated (%) in 355 mOsm/kg at 1 and 2 h of incubation were significantly (*p* < 0.05) higher than those in 290 mOsm media. The magnitude and duration of recovery of sperm progressive motility in 355 mOsm with pH 7.4 was correlated with the ejaculate rejection rate (R^2^ = 0.7). Using this information, the bulls were divided into good (n = 5) and poor (n = 5) osmo-adapters. The osmo-responsive genes such as *NFAT5, HSP90AB1, SLC9C1, ADAM1B* and *GAPDH* were upregulated (*p* < 0.05) in the sperm of good osmo-adapters. The study suggests that sperm are prepared for the osmotic and pH challenges in the female reproductive tract and the osmoadaptive ability is associated with ejaculate quality in bulls.

## Introduction

In mammals, sperm are exposed to gradients of osmolality and pH while traveling in the male and female reproductive tracts. In bovine, the osmolality of the epididymis is 350 mOsm/kg and seminal plasma is 280–300 mOsm/kg^1^, whereas the uterine and oviduct osmolality is hyperosmotic (350–355 mOsm/kg)^2^. These physiological stressors necessitate the sperm to undergo maturation in the epididymis and desirable functional changes in-terms of capacitation and subsequently the acrosome reaction before they come in contact with oocyte.

Sperm motility has been highly compromised in hypoosmotic (below 150 mOsm/kg) and hyperosmotic (500 mOsm/kg and above) solutions^3^. However, in vitro fertilization was achievable over a wide range of osmolality from 308 to 372 mOsm/kg in the mouse and 292 to 392 mOsm/kg in the hamster^4^. In bovine, an insignificantly higher percentage of in vitro fertilization has been achieved in hyperosmotic (340 mOsm/kg) over isosmotic media^5^. The osmotic stress is well known to induce mitogen-activated kinases (MAPK) which are intimately associated with the sperm capacitation process^6^. The kinases mediate post-translational modification of proteins in sperm which are involved in the capacitation, acrosome reaction, and fertilization processes^7^.

Sperm volume regulation in response to the osmotic stress is indispensable to achieve fertilization^8^. There has been a strong positive correlation of sperm osmo-tolerance ability with fertility and fecundity^9,10^. Despite the existence of osmotic stress in the tightly regulated reproductive tract micromilieu, the information on the osmo-adaptive ability of sperm and the possible impact on their functional competence have not been studied in detail.

In bovine, pH of the seminal plasma and uterus is 6.8—6.9, whereas the pH of the oviduct is 7.4^11,12^. Sperm metabolic activities have been reported to be maximum within the physiological pH ranges of 6.9 to 7.4^13,14^. Sperm are quiescent in acidic epididymal plasma^15,16^, but gain motility in comparatively alkaline seminal plasma^17^. The gradual exposure of sperm towards alkaline pH during the course of fertilization activates capacitation and hyperactivation by triggering the signaling events associated with tyrosine phosphorylation, cholesterol efflux, calcium influx and alkalization of cytoplasmic pH^18,19^.

Sperm are transcriptionally and translationally silent, but they are endowed with the necessary biomolecules including transcripts and proteins for accomplishing fertilization. Sperm retained RNA indicates the transcriptional history of spermatogenesis and responses to the external stimuli with the limited transcriptional activities. Hence assessing the relative expression levels of stress associated genes in fresh sperm may shed insight into the sperm preparedness towards osmotic and pH stress adaptation ability. The osmo-responsive genes that are involved in various functions such as oxidative phosphorylation (*MT-ND2* and *COX1*), glycolysis (*ENO1* and *GAPDH*), and calcium ion and hyperosmotic sensor (*EFHD1/mitocalcin*) activities^24^ regulate sperm function and fertilization. In addition, studying the expression levels of the genes associated with other stress responses including chaperone regulating signaling events (*HSP90AB1*), transcriptional regulation of osmo-protective and inflammatory genes (*NFAT5*), sperm-specific intracellular pH regulator (*SLC9C1*), and a sperm egg fusion disintegrin and metalloprotease (*ADAM1B*)^20–23^ in sperm may provide information on the osmo-adaptation ability of an ejaculate.

Continuous monitoring of the adaptation dynamics of the sperm in a simulated microenvironment of the female reproductive tract may provide information for developing an effective method for selection of fertile ejaculate for artificial breeding. Hence, the study was designed to understand the preparedness of sperm for adapting to the prevailing stressors in the female reproductive tract with an aim i) to analyze the adaptive ability and functional changes in sperm attributes at the physiological ranges of osmotic and pH stressors, and ii) to assess the expression levels of stress regulatory genes.

## Results

### Sperm kinematics and head area

**Figure 1:**
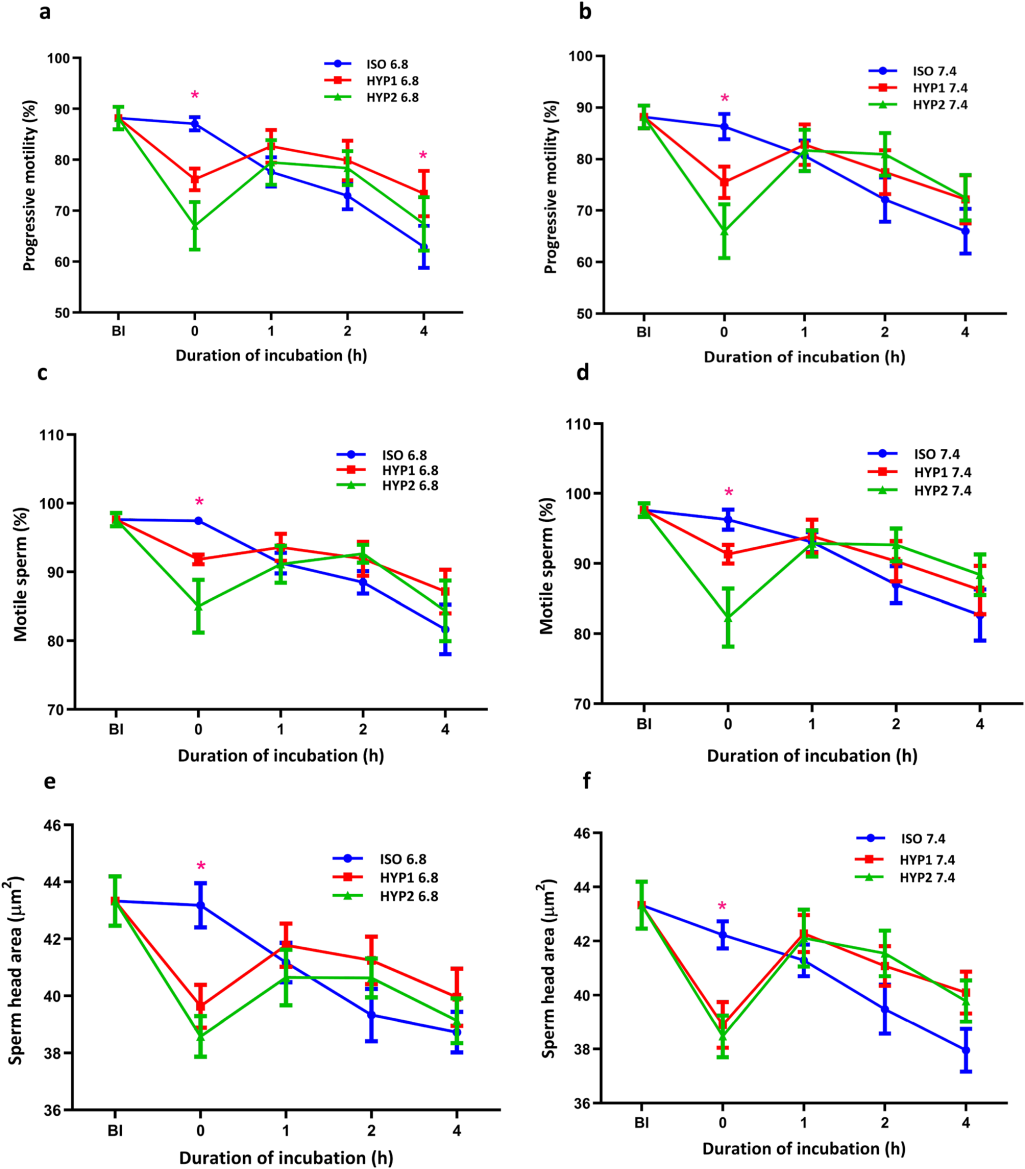
Effect of osmolality and pH on bovine sperm progressive, total motility and head area in vitro. The hyperosmotic (HYP1) uterine (**a**) and oviduct (**b**) environment significantly (*p* < 0.05) decreased the progressive motile sperm at 0 h and regained the same at 1 h of incubation. The progressive motility (**a**) was significantly higher at 4 h of incubation in HYP1 as compared to ISO only at pH 6.8. Though the total motility differed significantly at 0 h of incubation in both pH, no significant difference was observed in other time periods of both pH 6.8 (**c**) and 7.4 (**d**). The hyperosmotic (HYP1) uterine (**e**) and oviduct (**f**) environment significantly (*p* < 0.05) decreased sperm head area at 0 h and regained at 1 h of incubation. The interaction between osmolality and incubation time was significant (*p* < 0.05) for sperm progressive motility, total motility and head area. *represent the effects of osmolality, differ significantly (*p* < 0.05) at a particular time point. (ISO: 290 mOsm/kg; HYP1: 355 mOsm/kg; HYP2: 420 mOsm/kg; BI:Before incubation).

The average osmolality (mOsm/kg) of seminal plasma (*n* = 12) was 292 ± 2.72 ranging from 277 to 313. The pH of the bull semen (*n* = 12) was 6.75 ± 0.05 ranging from 6.5 to 7.0. Immediately (0 h) after the addition of sperm to the media, the percent progressive and total motile sperm significantly (*p* < 0.05) decreased in the hyperosmotic than the isosmotic media at both pH of 6.8 (Fig. 1a and c) and 7.4 (Fig. 1b and d). Subsequently, a significant (*p* < 0.05) recovery in progressive motility was observed in hyperosmotic media at 1 h as compared to 0 h in pH 6.8 (Fig. 1a). The percent progressive motility was significantly (*p* < 0.05) higher at 4 h in 355 mOsm/kg (HYP1) as compared with 300 mOsm/kg (ISO) media at uterine pH, 6.8 (Fig. 1a). Similarly, a significant (*p* < 0.05) decrease in sperm head area (µm^2^) was observed on immediate exposure (0 h) to hyperosmotic stress. Subsequently, a significant increase in sperm head area (µm^2^) was observed at 1–2 h indicating a regulatory volume increase. However, the recovery rate or adaptation ability was comparatively lower in HYP2 (420 mOsm/kg) than that of HYP1 at both pH (Fig. 1e and 1f).

**Figure 2:**
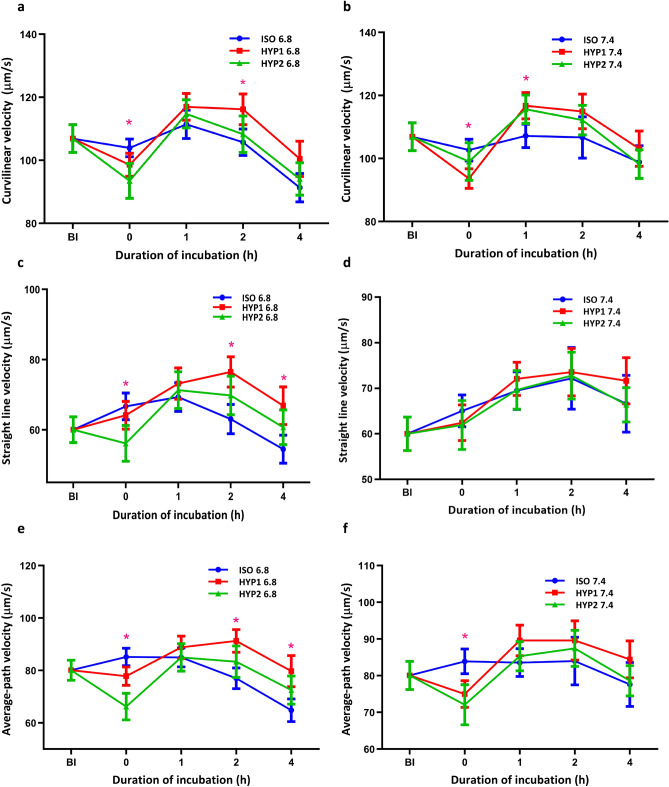
Effect of osmolality and pH on bovine sperm velocities (µm/s) incubated in vitro. The hyperosmotic (HYP1) uterine environment (pH 6.8) significantly (*p* < 0.05) increased VCL (**a**), VSL (**c**), VAP (**e**), post osmo-adaptation at 2 h of incubation. However, the hyperosmotic (HYP1) oviduct environment (pH 7.4) did not significantly affect VCL (**b**), VSL (**d**), VAP (**f**) at 2 h of incubation. The interaction of osmolality with incubation time was significant (*p* < 0.05) for VCL, VSL and VAP. The interaction between pH and incubation time was significant (*p* < 0.05) for VSL and VAP. *represent the effects of osmolality, differ significantly (*p* < 0.05) at a particular time point. (ISO: 290 mOsm/kg; HYP1: 355 mOsm/kg; HYP2: 420 mOsm/kg; BI:Before incubation; VCL: Curvilinear velocity; VSL: Straight line velocity; VAP: Average path velocity).

The curvilinear velocity (µm/s), straight-line velocity (µm/s) and average path velocity (µm/s) of sperm in HYP1 at pH 6.8 were significantly (*p* < 0.05) higher as compared with ISO at pH 6.8 at 2 and 4 h of incubation (Fig. 2). The ALH (µm/sec) and BCF (Hz) were significantly (*p* < 0.05) higher in HYP1 than ISO (pH 6.8) media at 1 h of incubation (Supplementary Fig. S1).

### Sperm capacitation status

**Figure 3:**
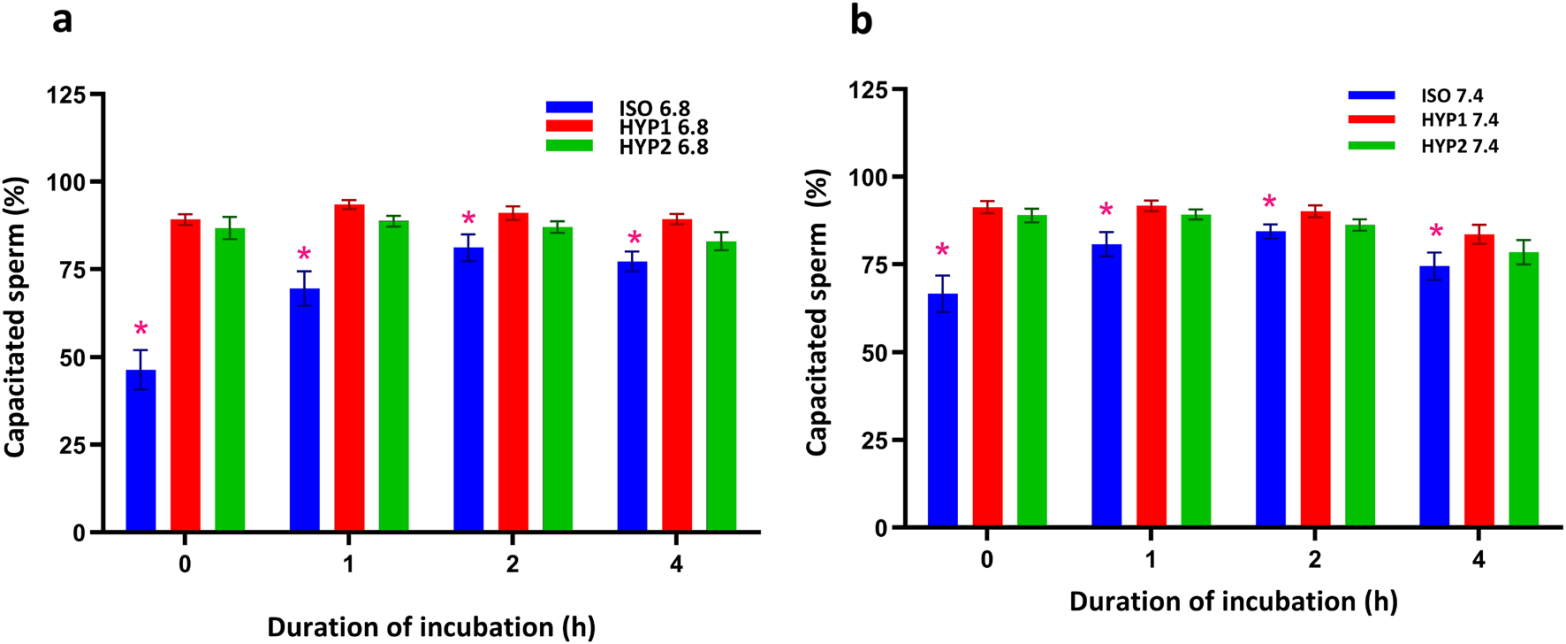
Effect of osmolality and pH on capacitation status in bovine sperm incubated in vitro. The hyperosmotic (HYP1) uterine (**a**) and oviductal (**b**) environment significantly (*p* < 0.05) increased capacitation of sperm. The interaction of pH with osmolality and incubation time was significant (*p* < 0.05) for capacitation status. *represent the effects of osmolality, differ significantly (*p* < 0.05) at a particular time point. (ISO: 290 mOsm/kg; HYP1: 355 mOsm/kg; HYP2: 420 mOsm/kg; BI: Before incubation).

The osmolality and pH had a significant (*p* < 0.001) interaction effect on sperm capacitation status. The hyperosmotic media at pH 6.8 (Fig. 3a) and pH 7.4 (Fig. 3b) induced capacitation in a significantly higher percentage of sperm on immediate exposure (0 h) than that of isosmotic media of respective groups (Supplementary Fig. S2). Further, the pH also influenced the capacitation process in sperm as the percentage of sperm capacitated in the pH 7.4 was significantly (*p* < 0.001) higher in the isosmotic media (ISO 7.4) at 0 and 1 h as compared to pH 6.8.

### Sperm functional membrane integrity and acrosome integrity

**Figure 4:**
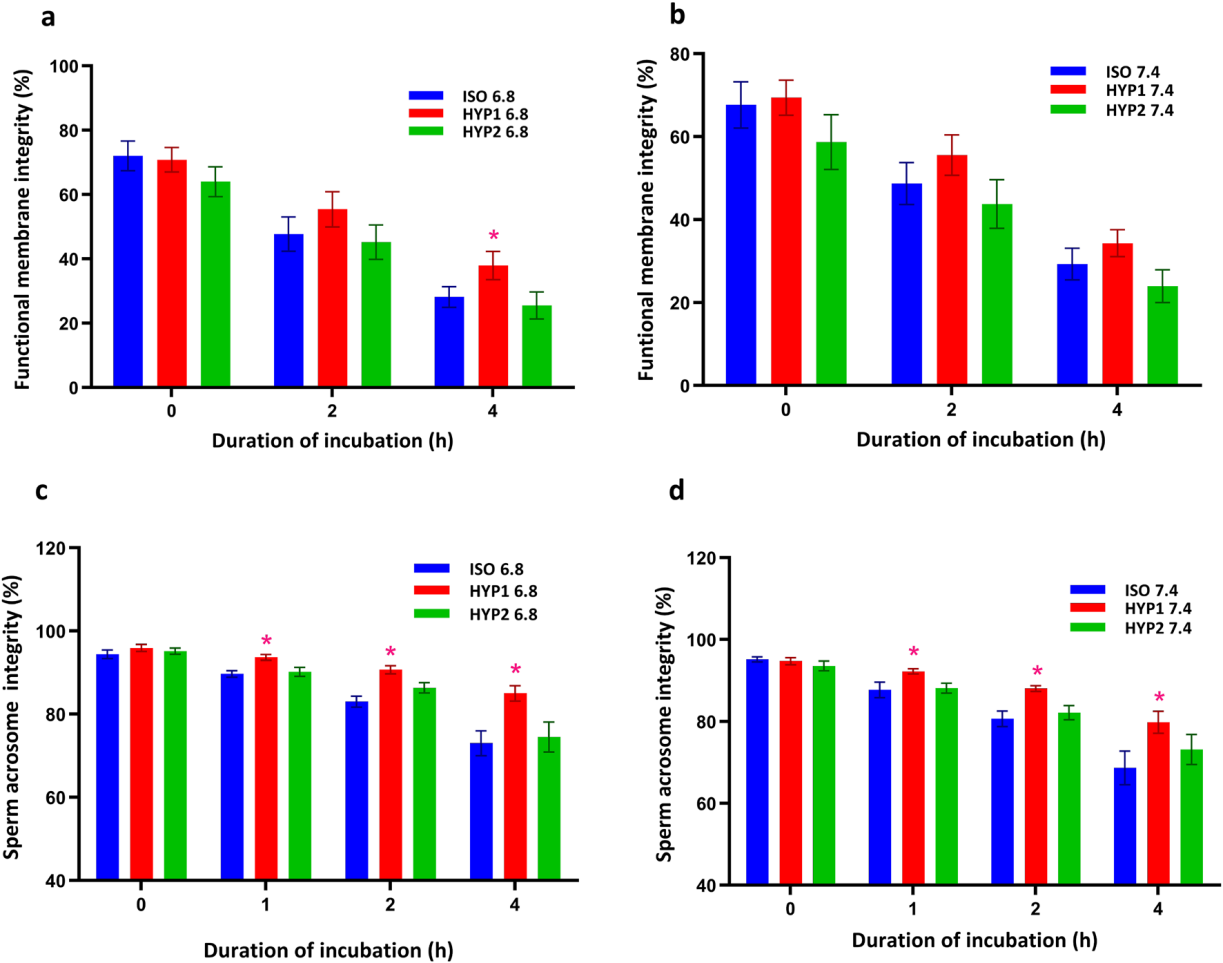
Effect of osmolality and pH on functional membrane integrity (**a**, **b**) and acrosome integrity (**c**, **d**) in bovine sperm incubated in vitro. The hyperosmotic (HYP1) uterine (**a**) environment significantly (*p* < 0.05) protected the functional membrane integrity at 4 h of incubation as compared to isosmotic condition. The acrosome integrity was also maintained significantly higher in sperm incubated at 355 mOsm in both pH 6.8 and 7.4 from 1 h. The interaction between osmolality and incubation time was significant (*p* < 0.05) for sperm acrosome integrity. *represent the effects of osmolality, differ significantly (*p* < 0.05) at a particular time point. (ISO: 290 mOsm/kg; HYP1: 355 mOsm/kg; HYP2: 420 mOsm/kg; BI: Before incubation).

The uterine osmolality (HYP1, 355 mOsm) with pH of 6.8 significantly (*p* < 0.05) protected the functional membrane integrity as compared to ISO and HYP2 media at 4 h of incubation (Fig. 4a). However, the oviductal osmolality (HYP1, 355 mOsm) with a pH of 7.4 did not protect functional membrane integrity as compared to ISO and HYP2 media (Fig. 4b). The acrosome intact sperm (%) were significantly higher (*p* < 0.05) in HYP1 at pH of the uterus (Fig. 4c) and oviduct (Fig. 4d) from 1 h of incubation.

### Sperm viability, mitochondrial membrane potential, and chromatin distribution

The viability of the sperm was significantly (*p* < 0.05) affected immediately after exposure to hyperosmotic (HYP1) medium with both pH 6.8 and 7.4. Thereafter, sperm viability was significantly (*p* < 0.05) higher in HYP1 as compared to isosmotic media (Table 1). The mitochondrial membrane potential was also significantly (*p* < 0.05) protected in hyperosmotic uterine pH (HYP1, 6.8) at 2 and 4 h of incubation as compared to both isosmotic and HYP2 media. The chromatin distribution was not influenced by pH and osmolality in our study.

**Table 1:** Influence of osmolality and pH on viability and mitochondrial membrane potential in bovine sperm incubated in vitro (Mean ± SEM). DOI: Duration of incubation; ISO: 290 mOsm/kg; HYP1: 355 mOsm/kg; HYP2: 420 mOsm/kg; BI: Before incubation.

Parameters	DOI	Uterine pH 6.8			Oviduct pH 7.4		
		ISO	HYP1	HYP 2	ISO	HYP1	HYP 2
Sperm viability (%)	BI	93.1 ± 1.0^X^	93.1 ± 1.0^X^	93.1 ± 1.0^X^	93.1 ± 1.0^X^	93.1 ± 1.0^X^	93.1 ± 1.0^X^
	0 h	92.4 ± 1.32^aX^	87.3 ± 1.17^bY^	83.1 ± 4.10^bY^	91.3 ± 1.90^aX^	87.2 ± 1.47^bY^	86.2 ± 3.89^bY^
	1 h	83.3 ± 2.30^aY^	88.4 ± 2.80^bY^	84.6 ± 3.53^abY^	86.5 ± 3.10^X^	87.6 ± 2.80^Y^	86.0 ± 3.50^Y^
	2 h	78.2 ± 2.93^aY^	84.7 ± 2.93^bY^	84.2 ± 2.78^bY^	77.3 ± 2.62^Y^	82.5 ± 2.89^Y^	85.1 ± 3.12^Y^
	4 h	70.7 ± 3.38^aZ^	78.9 ± 2.37^bZ^	74.3 ± 2.52^abZ^	71.2 ± 3.79^Y^	77.5 ± 3.54^Y^	78.2 ± 4.15^Y^
Mitochondrial membrane potential (%)	0 h	85.5 ± 2.68^X^	85.3 ± 2.97^X^	82.1 ± 2.96^X^	82.9 ± 2.90^X^	85.5 ± 3.54^X^	83.2 ± 3.33^X^
	2 h	79.8 ± 3.78^aX^	86.6 ± 2.86^bX^	77.8 ± 5.75^X^	83.3 ± 4.21^X^	83.4 ± 4.31^X^	76.9 ± 3.91^X^
	4 h	69.2 ± 4.92^aY^	77.9 ± 4.75^bX^	66.8 ± 6.86^X^	77.1 ± 5.05^X^	79.8 ± 5.14^X^	66.8 ± 5.79^Y^

Values with superscript bearing ^a,b^ within a row for a particular time point and pH differ significantly (*p* < 0.05). Values with superscripts bearing ^X,Y,Z^ within a column for a particular parameter differ indicating a significant effect of time (*p* < 0.05).

The interaction effect of osmolality by incubation time was significant (*p* < 0.05) for sperm viability and mitochondrial membrane potential.

### Relationship between sperm osmo-susceptibility and ejaculate quality

The study results revealed a significant (*p* < 0.05) negative correlation of ejaculate rejection rate with the sperm mitochondrial membrane potential (-0.67 and -0.73), functional membrane integrity (-0.60 and -0.59), and acrosome integrity (-0.72 and -0.77) in the HYP1 with a pH of 6.8 and 7.4, respectively at 1 h as compared to the other media (Supplementary Table S1).

**Figure 5:**
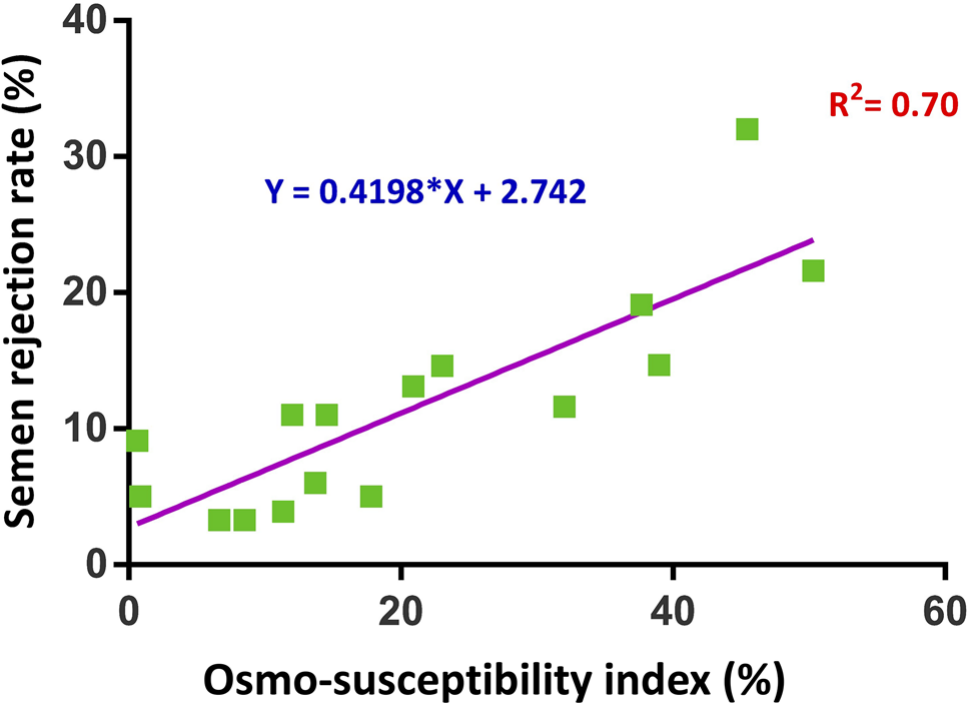
Relationship between the ejaculate rejection rate with osmo-susceptibility index. The coefficient of determination of the regression equation (R^2^) was 0.70.

The deterioration in progressive motility was associated with lower sperm osmo-adaptation ability. Based on this information, osmo-susceptibility indices were developed to assess semen quality. Out of the six osmo-susceptibility indices developed, the osmo-susceptibility index showing a strong positive correlation (*r* = 0.71) with the ejaculate rejection rate (supplementary Fig. S3 and S4) was selected for the classification of bulls as good and poor osmo-adapters, and subsequent gene expression studies. The index at 18% cut-off had the maximum likelihood ratio of 6. At the chosen cut-off, the specificity was 83.3% and sensitivity was 100%. The linear regression equation had a significant coefficient of determination (R^2^) of 0.70 for the prediction of ejaculate rejection rate (Fig. 5). When the index was validated by including another set of animals (n = 6), the index had a high correlation (*r* = 0.79) with the ejaculate rejection rate. The obtained index was also significantly (*p* < 0.05) negatively correlated with the sperm progressive motility, total motility, mucus penetration, ALH, BCF, head area, functional membrane integrity and mitochondrial membrane potential, but positively correlated with the acrosome reaction (Supplementary Table S2).

### Expression of osmo-responsive genes in sperm

**Figure 6:**
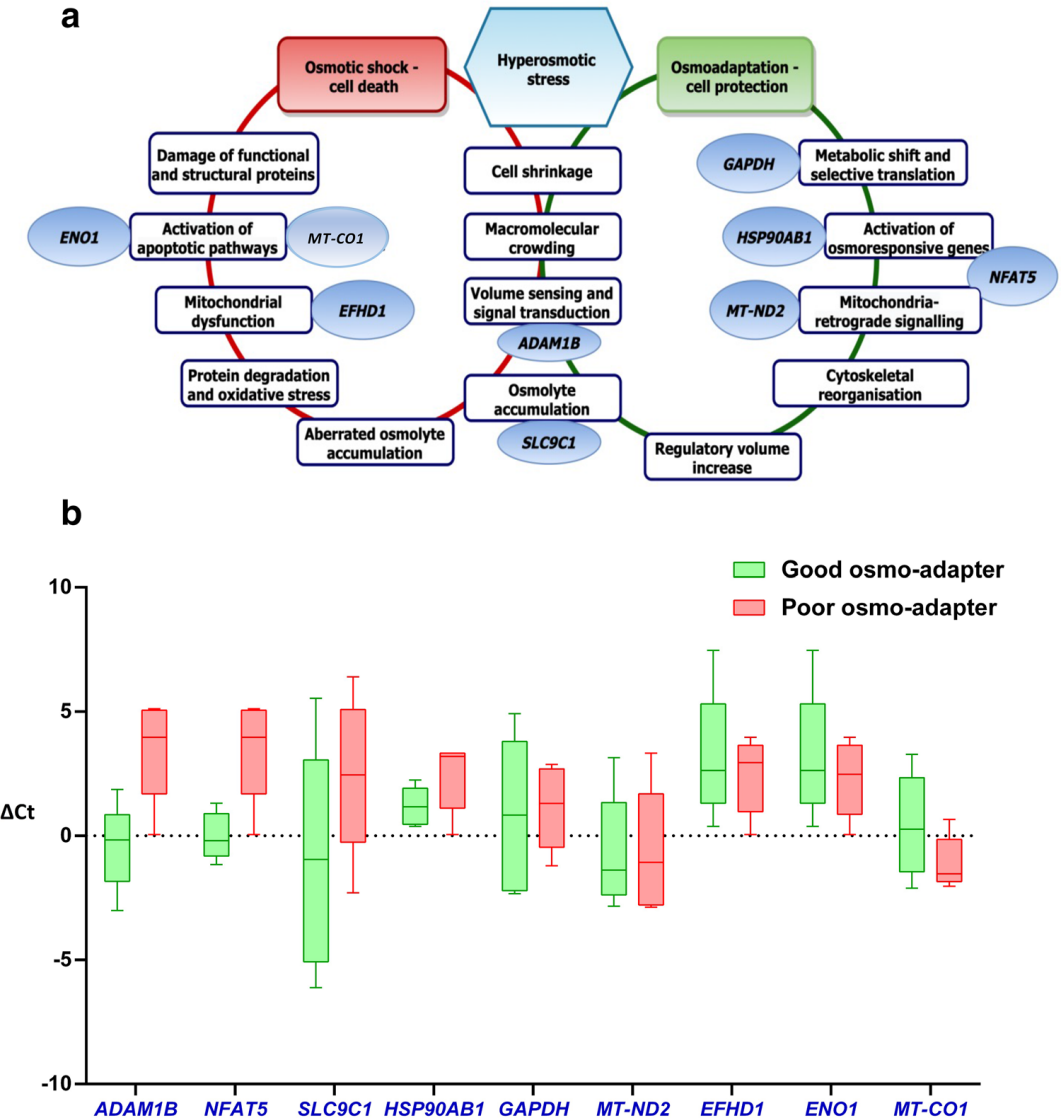
The osmo-responsive genes and their function pertaining to osmotic stress (**a**). The relative gene expression levels of osmo-responsive genes normalized to housekeeping gene *RPL 23* (ΔCt) in good and poor osmoadapters (**b**). The relative expression levels of the genes *NFAT5,* and *ADAM1B* were significantly (*p* < 0.05) higher in good osmo-adapter group as compared to poor osmoadapters.

In the present study, *RPL23* served as endogenous control in comparison with *PRM1* after analysing the expression levels of both genes in NormFinder (M value-0.53) and BestKeeper (correlation coefficient-0.87) softwares. The relative expression levels of the genes *NFAT5,* and *ADAM1B* were significantly (*p* < 0.05) higher in good osmo-adapters (Fig. 6). Importantly, the expression levels of *NFAT5* and *ADAM1B* genes were consistently non-detectable in the poor osmo-adapter group (n = 5). Similarly, the expression of *ENO1* and *EFHD1* genes were observed only in two out of five samples in poor osmo-adapter group. *HSP90AB1* and *GAPDH* expression levels were upregulated in the good osmo-adapter group with a fold change of 2.34 and 1.68, respectively. *MT-CO1* expression was higher in the poor osmo-adapter group than that of good osmo-adapter group. The relative expression levels of *MT-ND2* gene did not differ between the groups. The correlation of the expression levels of *NFAT5, ADAM1B, SLC9C1* and *HSP90AB1* were significantly (*p* < 0.05) positive, whereas MT*-CO1, EFHD1* and *ENO1* were significantly (*p* < 0.05) negative with sperm functional attributes in the present study (Supplementary Table S3).

## Discussion

Sperm are exposed to the inevitable osmotic and pH excursions in the reproductive tract during their quest to fertilize the ovum. Since bull sperm stay in the female reproductive tract for at least 6–12 h before accomplishing fertilization, the uterine and oviductal microenvironment play an important role in selecting the competent sperm for fertilization.

The study revealed that significantly higher percent of sperm were capacitated in uterine and oviduct hyperosmolality as compared to the isosmotic medium. In fact, the high ionic strength (HIS) medium or modified Brackett and Oliphant medium (BO) of 380 mOsm/kg has been used as a capacitation media for guinea pigs, rabbits, and later in bovine^26,27^. The high ionic nature of the medium was proposed to efficiently remove the decapacitating factor, cholesterol from the sperm surface. The hyperosmotic condition also enhances tyrosine phosphorylation of sperm proteins, a hallmark of sperm capacitation, and zona pellucida binding capacity of human sperm^28^.

The sperm motility and velocities were significantly affected upon exposure to osmotic stress. After adaptation, the progressive motility, VSL, and VAP improved with the recovery of sperm head area. Besides, the velocity regained during osmo-adaptation in the hyperosmotic medium at 2 h was significantly higher than 0 h as well as the corresponding time points in the control medium. A sharp increase in the sperm motility is often attributed as a characteristic feature of the capacitated sperm^29^. In the present study, the ALH and BCF also increased substantially at 1 h in the hyperosmotic medium and can be attributed to hyperactivation^30,31^. In addition, in this study lower level of bicarbonate (12 mM) was used in the medium whereas at least 25 mM bicarbonate was required for sperm hyperactivation^32^. The extracellular hyperosmolality resulting in cell shrinkage activates chloride bicarbonate exchanger favoring the influx of bicarbonate to regulate the volume^33^. In addition, the osmotic stress signaling in somatic cells is mediated by initial calcium influx^36^. Similarly, addition of sperm to the hyperosmotic medium, resulted in instantaneous calcium influx as evidenced from the appearances of CTC-pattern B, which is indicative of capacitation. Thus, the altered sperm kinematics during capacitation in the present study might be as a result of physiological osmotic stress imposed on sperm. The oviductal pH having a capacitating effect on the isosmotic medium has been widely accepted in many species^34,35^. The present study revealed significant interaction effect of osmolality and pH in regulating the sperm capacitation.

The uterine hyperosmolality significantly protected sperm viability both in terms of structural and functional membrane integrities, though the membrane integrity was reported to be optimally maintained between 200 and 300 mOsm/kg^3^. The variations between earlier observations and the present study may be also due to single time point evaluation, as the hyperosmolality mediated effect on sperm membrane integrities were evident only after 2 h of incubation. Furthermore, the hyperosmotic medium prevented the percentage of sperm undergoing acrosome reaction by protecting acrosome integrity. Hyperosmotic stress causes the cells to undergo actin polymerization and reorganization as a defense strategy resulting in protection of cell membrane, maintenance of cell shape, and promoting cell motility^37,38^. In sperm, actin polymerization occurs during capacitation and acrosome reaction^39^. In the present study, as the sperm were not challenged with acrosome reaction inducers, the higher acrosome integrity and longer sustenance of progressive motility in the hyperosmotic medium can also be attributed to the polymerization of actin in the sperm membrane^40^.

The mitochondria are the stress perceiving organelles and their crosstalk with the nucleus decide the fate of cell survivability^41^. The mitochondrial membrane depolarization and elevation of membrane potential in response to osmotic stress is a determinant of cellular adaptation^42^. The bovine sperm mitochondrial membrane potential tended to be higher in hyperosmotic uterine pH rather than oviductal pH indicating an efficient osmo-adaptation process at uterine pH. The present study suggests that the osmotic stress markedly influences the sperm functional attributes and thereby may influence male fertility.

Sperm functional attributes like functional membrane integrity, acrosome integrity and mitochondrial membrane potential in hyperosmotic media were more significantly correlated with ejaculate rejection rate than isosmotic media. This may be due to simulating the reproductive tract microenvironment, which is rather uncommonly used for bull fertility prediction tests. The progressive motility was initially hampered in hyperosmotic media due to the osmotic stress and it improved with the increase in the sperm head area. The recovery of progressive motility differed temporally and in magnitude between bulls; hence can be preferred as the suitable parameter representing sperm osmo-adaptation. Based on these observations, osmo-susceptibility indices were developed by calculating the loss of sperm progressive motility in hyperosmotic media having oviduct pH and osmolality. The regression equation suggests that 70% of the variations observed in the ejaculate rejection rate may be contributed by the sperm osmo-susceptibility.

Since the ejaculated sperm are transcriptionally and translationally silent, preparedness towards osmo-adaptation events were studied by assessing the expression levels of the stress-regulating genes in sperm. The expression levels of the osmo-responsive genes *NFAT5, HSP90AB1, SLC9C1, ADAM1B,* and *GAPDH* were upregulated, whereas *MT-CO1, ENO1,* and *EFHD1* were downregulated in the good osmo-adapters. *NFAT5* also known as tonicity responsive enhancer binding protein (TonEBP) acts as a transcription factor for several effectors of the osmo-adaptation process namely aquaporins, aldolase reductase, sodium myoinositol transporter^43^, taurine transporter, betaine transporter^44^, heat shock proteins^45^ and urea transporter^46^. In mice, *NFAT5* knockout resulted in embryonic mortality signifying its essential role in reproduction. NFAT5 protects the epididymal cells from hyperosmotic stress by regulating hypertonicity-induced genes expression^47^. The positive association of *NFAT5* expression level with sperm head area, mitochondrial membrane potential, percent hyperactive sperm and a strong negative correlation with semen rejection rate are evidence for *NFAT5* mediated osmo-adaptation regulation in sperm.

The study reveals that *ADAM1* and *SLC9C1* genes positively regulate sperm acrosome integrity*.* ADAM1 is a membrane-anchored protease that activates downstream kinases such as MAPK, ERK, and JUNK during osmotic stress^21^. These kinases mediate post-translational modification of sperm proteins during capacitation^48^. SLC9C1, sperm-specific sodium proton exchanger (sNHE) is an effector protein during osmotic stress and controls the accumulation of solute inside the cell to overcome cell shrinkage. SLC9C1 regulates sperm intracellular pH^49^ and cellular volume^50,51^. Moreover, *SLC9C1* knockout mice had absolute male infertility^52^. In addition, expression levels of *SLC9C1* were also negatively associated with the ejaculate rejection rate signifying the importance of this gene in promoting semen quality.

Heat shock proteins are molecular chaperones recruited under stress conditions to protect the cellular proteins^53^. HSP90 has been reported to regulate sperm motility, calcium influx, and capacitation process^54^. In this regard, the upregulation of *HSP90AB1* in the good osmo-adapter group suggests that HSP90AB1 may protect sperm proteins during osmotic stress and thereby positively influence sperm motility. Apart from these genes, *GAPDH* was 1.6 fold upregulated in the good osmo-adapter group. Under the osmotic stress conditions, the cellular metabolic reprogramming recruits the constitutive proteins for other cellular functions of necessity. Such multi-tasking proteins are called moonlighting proteins and GAPDH performs such functions under osmotic stress^55^.

A strong negative influence of *EFHD1* and *ENO1* with sperm motility and mitochondrial membrane potential is suggestive of their role on mitochondrial dysfunction and activation of apoptotic pathways in poor osmo-adapter semen^56,57^.

The study suggests that the uterine and oviductal osmolality though impede sperm motility immediately upon exposure, the sperm have the ability to overcome the stress. The hyperosmolality protects the structural, functional membrane and acrosomal integrities apart from favoring capacitation by promoting calcium influx within sperm. The reproductive tract osmotic stress and pH excursions are more of appreciative nature towards fertilization. Overall, upregulation of some of the stress-responsive genes including *HSP90AB1, SLC9C1, ADAM1B,* and *NFAT5* in good semen samples is suggestive of the preparedness of sperm to counter various stresses in the reproductive tract micromilieu.

## Materials and methods

ICAR-National Institute of Animal Nutrition and Physiology’s Institutional Animal Ethics Committee approved the present study (NIANP/IAEC/1/2020/10). All methods were carried out in accordance with relevant guidelines and regulations.

### Procurement and transport of semen sample

Fresh semen samples from Holstein Friesian (HF) bulls were procured from Nandini Sperm Station, Hessarghatta, Bengaluru. The ejaculates having at least 500 million sperm/ mL and 70% progressive motility were selected for the study. From each bull (n = 12), two ejaculates were collected and pooled to minimize the biological variation between the ejaculates. An aliquot of semen samples (n = 12) was diluted (1:1) in the isosmotic modified tris egg yolk extender (mTEY) and transported to the laboratory at 25–28 °C within 4 h. Another aliquot was immediately centrifuged at 10,000 g for 5 min at 4 °C and the seminal plasma was removed. The sperm pellet was resuspended in 1 ml of PBS (pH 7.4), snap frozen in liquid nitrogen and stored at -80 °C until RNA isolation^58^. The ejaculate rejection rate for each bull during the past one year was obtained from the semen station. The ejaculate with anyone of the following criteria was rejected: concentration < 500 million sperm/ml of ejaculate, mass motility < 3, or individual progressive motility < 70%.

### Experimental design

Tyrode basal medium (290 mOsm, pH 6.8) was considered as the isosmotic control medium. The desired hyperosmolarity 355 (hyperosmotic- test) and 420 (hyperosmotic-control) mOsm was obtained by adding 11.8 and 22.7 g of fructose per liter of medium, respectively. The fructose was preferred to increase the osmolality as it belongs to the class of organic and non-ionic osmolyte. Osmolality was measured using osmometer (OSMOMAT 3000, Gonotec, Germany), which calculates osmolality based on freezing point depression of a solution. In the test and control media, the pH was adjusted to 7.4 using 5 N sodium hydroxide and measured with digital pH meter (EuTech PH 700 benchtop pH meter, Qtech, India). Maintenance of osmolality after the pH adjustment was checked and confirmed.

In the laboratory, the diluted semen (100 μL) was layered over modified low bicarbonate Tyrode medium (mLBT, 1 mL) in a conical bottom microcentrifuge tube and centrifuged at 200 g for removing seminal plasma and egg yolk. After washing, the sperm pellet was resuspended in mLBT (100 µL) and concentration was measured. The semen sample (100 µL) containing about 100 million cells was transferred to each control and test media (900 µL) and incubated in a water bath at 37 °C for 4 h. At 0, 1, 2 and 4 h of incubation, sperm functional parameters such as sperm viability, kinematics, sperm subpopulation positive for functional membrane integrity, mitochondrial membrane potential, chromatin distribution, capacitation reaction and acrosome integrity were evaluated (Supplementary data I).

### Sperm functional attributes

Sperm viability was assessed using eosin and nigrosin stain^59^. Sperm that appear unstained were considered live and partially or completely stained with pink were considered dead. Sperm kinematic parameters were analyzed using a computer-aided sperm analyzer (CASA; Sperm Class Analyser, Version 6.4, Microptic SL, Spain)^60^. Hypo-osmotic swelling –Giemsa test (HOS-G) was carried out to assess the subpopulation of sperm positive for functional membrane integrity^61^. Sperm chromatin distribution was assessed by Feulgen’s staining method^61^. Sperm mitochondrial membrane potential was evaluated using mitochondria-specific cationic fluorophore JC-1(5,5`,6,6`-tetrachloro-1,1`,3,3`-tetraethyl benzimidazolyl carbocyanine iodide) and examined under fluorescent microscope^61^. Fluorescein isothiocyanate with Pisum Sativum Agglutinin (FITC – PSA) was used to assess acrosome integrity^62^. For each of these tests, a minimum of 200 sperm was counted and analyzed.

Capacitation status of sperm was assessed using chlortetracycline assay^63^ with minor modifications. On a clean grease-free glass slide, equal volume (5 µL) of semen and chlortetracycline (0.39 mg/ml) were added, mixed well and allowed to react in dark for a few seconds. Then, glutaraldehyde (0.5 µL of 0.6%) was added and smeared. Antifade agent, DABCO- 1,4 diazo bicyclo (2,2,2) octane was added over the smear and covered with coverslip. A minimum of 100 cells was counted under a 100 × epifluorescence microscope (Nikon Eclipse 80i, Nikon, Japan) with an excitation filter of 510–560 nm and an emission filter of 505 nm.

### Relationship between sperm osmo-adaptation ability and ejaculate quality

The progressive motility of sperm obtained by CASA was used for computing the osmo-adaptation ability. The loss of progressive motility (PM) at different time points was calculated as follows:

- $${\text{Percent}}\;{\text{motility}}\;{\text{loss}}\;{\text{at}}\;1\;{\text{h }} = { }\frac{{\left( {{\text{ PM \% }}\;{\text{before}}\;{\text{incubation}}{-}{\text{PM \% }}\;{\text{in}}\;{\text{HYP }}1\;{\text{at}}\;1\;{\text{h}}} \right)}}{{{\text{PM\% }}\;{\text{before}}\;{\text{incubation }}\left( {{\text{BI}}} \right)}} \times 100$$
- $${\text{Percent}}\;{\text{motility}}\;{\text{loss}}\;{\text{at}}\;{ }4\;{\text{h }} = \frac{{\left( {{\text{ PM\% }}\;{\text{before}}\;{\text{ incubation}}{-}{\text{ PM\% }}\;{\text{in}}\;{\text{HYP }}1\;{\text{at}}\;{ }4\;{\text{h}}} \right)}}{{{\text{PM\% }}\;{\text{before}}\;{\text{incubation}}}} \times 100$$

The osmo-susceptibility indices, OSI1 (percent loss of progressive motility at 1 h in HYP1 media, 355 mOsm with pH, 6.8), OSI2 (percent loss of progressive motility at 4 h in HYP1 media, 355 mOsm with pH, 6.8) and OSI3 (sum of percent loss of progressive motility at 1 and 4 h in HYP 1 media with pH, 6.8) were calculated. Similarly, osmo-susceptibility indices, OSI4 (percent loss of progressive motility at 1 h in HYP1 media with pH, 7.4), OSI5 (percent loss of progressive motility at 4 h in HYP1 media with pH, 7.4) and OSI6 (sum of percent loss of progressive motility at 1 and 4 h in HYP1 media with pH, 7.4) were calculated.

The efficiency of different osmo-susceptibility indices was analyzed by correlating the indices with the fresh semen ejaculate rejection rate (Supplementary Fig. S3 and S4). Since OSI6 had the highest positive correlation with fresh semen ejaculate rejection rate, the optimal cut-off value of 18% was arrived based on receiver operating characteristic curve analysis. The semen quality prediction ability of the index was determined using the regression analysis and the model was also validated in randomly selected another set of animals (n = 6) of known rejection rate (Supplementary Table S4).

### Sperm osmo-regulation associated genes expression

Based on the chosen cut-off in the regression model, the semen samples were classified as good (n = 5) and poor (n = 5) osmo-adapters and the differential expression levels of osmo-responsive genes in sperm were studied between groups. This was used to assess the adaptive ability of sperm to meet the osmolarity changes in the female microenvironment.

The genes relevant to osmo-adaptation related sperm function were selected based on a literature survey (Fig. 6). The primers for the osmo-responsive genes *NFAT5*^20^, *ADAM1B*^21^*, SLC9C1*^22^*, HSP90AB1*^23^*, ENO1, EFHD, MT-ND2* and *GAPDH*^24^, *MT-CO1*^25^ were designed using Primer3Plus software (Table 2).

**Table 2:** The details of the Primers used for gene expression studies in the study.

Primer ID	Primer	Primer sequence (5′ to 3′)	Primer length (bp)	Product size (bp)	NCBI accession number
*SLC9C1*	Forward	GGAACGCCTCGAATAAGCCT	20	149	XM_024994321.1
	Reverse	TCAGCTCAAAGTTGCTCCCT	20		
*HSP90AB1*	Forward	GTGACGATCTCCAACAGGCT	20	213	NM_001079637.1
	Reverse	GTCGTTTTTGTCCGCCTCTG	20		
*EFHD1*	Forward	AACGTGCCTCTACTTGGCAG	20	145	NM_001075832.1
	Reverse	TTAACATCACTGGCCTCCCG	20		
*ENO1*	Forward	ATGTCACCGAGCAGTGTGAG	20	212	NM_174049.2
	Reverse	GATACTTGGTGGGAGCGAGG	20		
*NFAT5*	Forward	ACCTCTTCCAGCCCTACCAT	20	170	XM_002694839.6
	Reverse	AAGACTGTGTGCCTCTTCGG	20		
*MT-ND2*	Forward	TCTCAGGCCAATGAACCGTA	20	127	NC_006853.1:4266–5307
	Reverse	ATGCCCTGTGTTACTTCTGGG	21		
*MT-CO1*	Forward	GTAACCGCACACGCATTTGT	20	217	NC_006853.1:5687–7231
	Reverse	GGTACACGGTTCAGCCTGTT	20		
*ADAM1B*	Forward	GAGTGGGAATGACAGGCTCA	20	119	NW_020192236
	Reverse	TGACAGAATCCCTCCTCCTAGT	22		
*PRM1*	Forward	AAGATGTCGCAGACGAAGGAG	21	222	NM_174156.2
	Reverse	GTGGCATTGTTCGTTAGCAGG	21		
*RPL23*	Forward	CAGCGGTGGTAATTCGACAAC	21	116	NM_001035014.2
	Reverse	GGCGGAACCTTTCATCTCG	19		
*GAPDH*	Forward	CTGAGGACCAGGTTGTCTCCTG	22	141	NM_001034034.1
	Reverse	CCCTGTTGCTGTAGCCAAATTC	22		
*CDH1*	Forward	CTGCATTCCTGGCTTTGGTG	20	171	NM_001002763.1
	Reverse	GTAAGCACGCCATCTGTGTG	20		
*CKIT*	Forward	GAATAGCTGGCATCAGGGTG	20	224	AF263827.1
	Reverse	CCAGATCCACATTCTCTCCATC	22		
*PTPRC*	Forward	TGGACGAAATTGCATCCCTCAGGA	24	237	NM_174156.2
	Reverse	RTGGTCAGGACGTTTACAGCTCACA	24		

### RNA isolation and real time-PCR

Total RNA was extracted from sperm as per the established protocol^58^. Briefly, the snap frozen semen samples were thawed and resuspended in 1 ml of PBS (pH 7.4). The semen sample (1 mL) was layered over 4 mL of 50% Bovipure gradient solution in 15 mL conical bottom centrifuge tubes and centrifuged at 200 g for 20 min at room temperature (28 °C). The sperm pellet was re-suspended in 10 mL of PBS and washed by centrifugation at 700 g for 5 min at 4 °C. Then the sperm pellet was again re-suspended in 1 mL of PBS and sperm concentration was measured using a hemocytometer. Sperm (30—40 million cells) was subjected to double lysis and RNA extraction was carried out using the kit (PureLink RNA mini kit, Invitrogen, USA)^58^. The quality of the RNA was assessed using a spectrophotometer (NanoDrop, ND- 1000, Thermo Scientific, USA) by analyzing the absorbance ratios of 260/280 and 260/230. To remove the genomic DNA contamination, the isolated RNA was subjected to DNase treatment (TURBO DNA-free kit, Ambion, Life Technologies, USA). The total RNA was quantified using fluorometer (Qubit 4.0, Invitrogen). The RNA free from genomic DNA was subjected to complementary DNA (cDNA) synthesis using the first-strand cDNA synthesis kit (SuperScript IV, Invitrogen, USA). An equal quantity (20 ng) of RNA from each sample was used for cDNA synthesis. For the detection of RNA from other contaminating cells, the cDNA samples were tested with cell-specific gene primers, namely *CDH1* (Epithelial cadherin) for somatic cells, *CKIT* (Kit oncogene) for germ cells and *PTPRC* (Protein tyrosine phosphatase receptor type C) for leucocytes using qPCR (Table 2). *RPL23* and *PRM1* were compared for the suitability to consider as an endogenous control using NormFinder and BestKeeper tools. The gene with the best stability, *RPL23* was selected for normalizing the expression levels of sperm transcripts. The genes were amplified for 40 cycles and the unique product was confirmed by melt curve analysis. The product size was verified using agarose gel (2.0%) electrophoresis. Relative gene expression levels were computed using the 2^-ΔΔCt^ method^64^. The undetermined values in qPCR were replaced with maximum possible Ct value of 35 for statistical analysis for calculating fold change^65^.

### Statistical analyses

The percentage data were arcsin transformed before subjecting to the statistical analysis. A mixed model 2 × 3 × 5 factorial ANOVA with repeated measures was used to assess the treatment effects of pH (6.8 and 7.4), osmolarity (290, 355 and 420 mOsm/kg) and incubation time (before incubation, 0, 1, 2 and 4 h) on sperm kinematics, head area, membrane integrities, capacitation status, mitochondrial membrane potential, and chromatin distribution variables. As a first step, effects of interactions between treatments were examined. If not, the main effects for osmolality and pH were investigated. When the F value was significant, Tukey’s post-hoc analyses were conducted to determine pairwise differences. Analyses were carried out using IBM SPSS version 20 and GraphPad prism 6. The student’s t-test was used for analyzing the significant differences in sperm functional parameters and relative gene expression (∆Ct) between good and poor osmo-adapters. The capability of osmo-susceptibility index in predicting the ejaculate rejection rate was evaluated using ROC curve analysis. Pearson correlation was used for assessing the relationship between the osmo-susceptibility indices with ejaculate rejection rate. All the values were presented as mean ± SEM. The significance level was set at *p* ≤ 0.05.

## Supplementary Information

Supplementary Information 1.

Supplementary Information 2.

Supplementary Information 3.

Supplementary Information 4.

Supplementary Information 5.

## Data Availability

All data generated or analyzed during this study are available from the corresponding author on reasonable request.
