# Supplementary material for: Sperm preparedness and adaptation to osmotic and pH stressors relate to functional competence of sperm in Bos taurus
Source: Sci Rep. 2021 Nov 19;11:22563. doi: 10.1038/s41598-021-01928-6 (PMC8604908; doi:10.1038/s41598-021-01928-6)
Supplement: Supplementary file 1 — Supplementary Information 1. [file 41598_2021_1928_MOESM1_ESM.docx]

**Supplementary Information**

**Sperm preparedness and adaptation to osmotic and pH stressors relate to functional competence of sperm in *Bos taurus***

Maharajan Lavanya^a,b^, Santhanahalli Siddalingappa Archana^a^, Divakar Swathi^a^, Laxman Ramya^a^, Arunachalam Arangasamy^a^, Balakrishnan Binsila^a^, Arindam Dhali^c^, Narayanan Krishnaswamy^d^, Sanjay Kumar Singh^b^, Harendra Kumar^b^, Muniandy Sivaram^e^, Sellappan Selvaraju^a,*^

**Results**


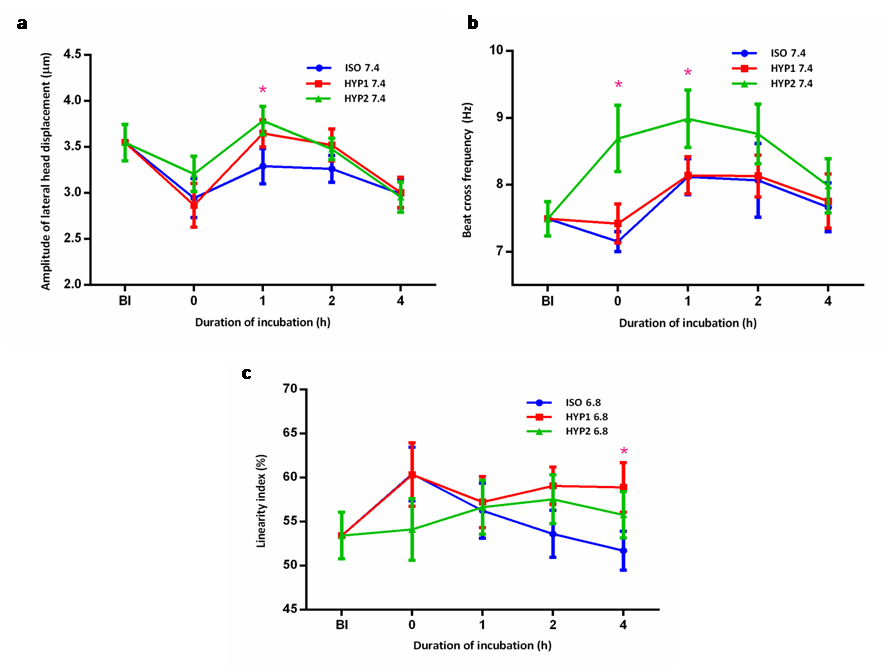


**Supplementary figure S1: Effect of osmolality and pH on sperm motility characteristics (BCF, ALH) in bovine sperm incubated *in vitro*. The hyperosmotic (HYP1 oviductal environment significantly (*p*<0.05) increased ALH (a), BCF (b) at 1 h. (ISO: 290 mOsm/kg; HYP1 : 355 mOsm/kg; HYP2 : 420 mOsm/kg; BI:Before incubation)**

**
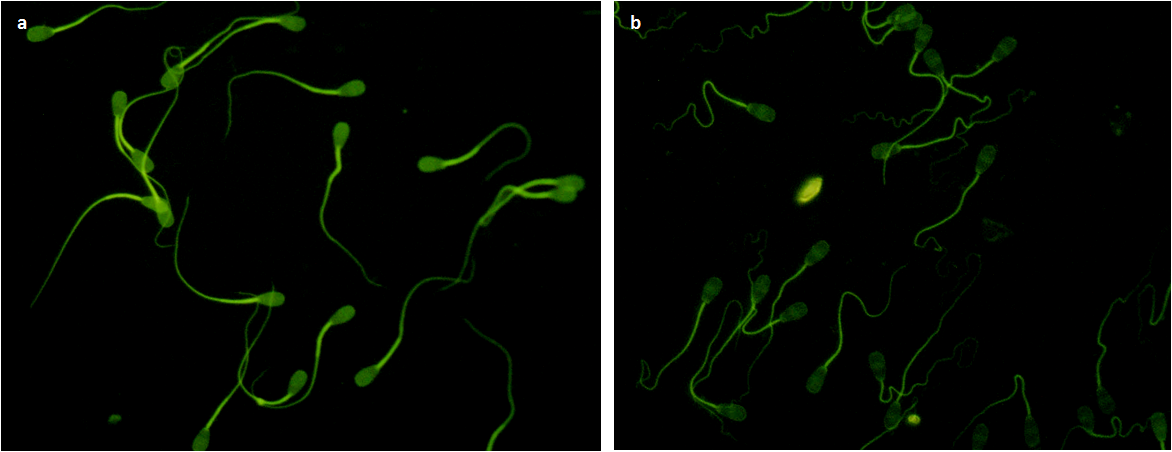
**

**Supplementary figure S2: Effect of osmolality on sperm capacitation. Capacitation was assessed by chlortetracycline assay. Representative images indicate (a) non-capacitated sperm in the isosmotic (ISO 6.8) medium and (b) capacitated sperm in the hyperosmotic (HYP1 6.8) media at 0 h of incubation.**


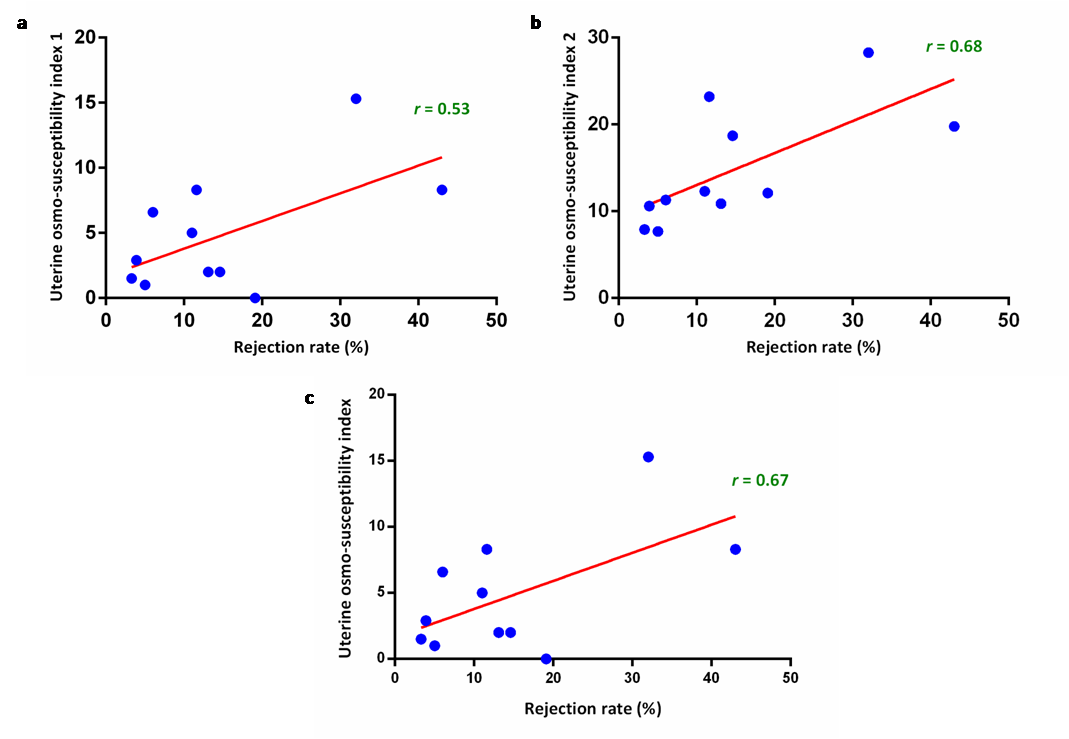


**Supplementary figure S3: Correlation between predicted uterine osmo-susceptibility indices (uterine osmo-susceptibility indices at 1 h (UTI 1- a), at 4 h (UTI 2 - b) and total score (UTSI - c)) with ejaculate rejection rate.**


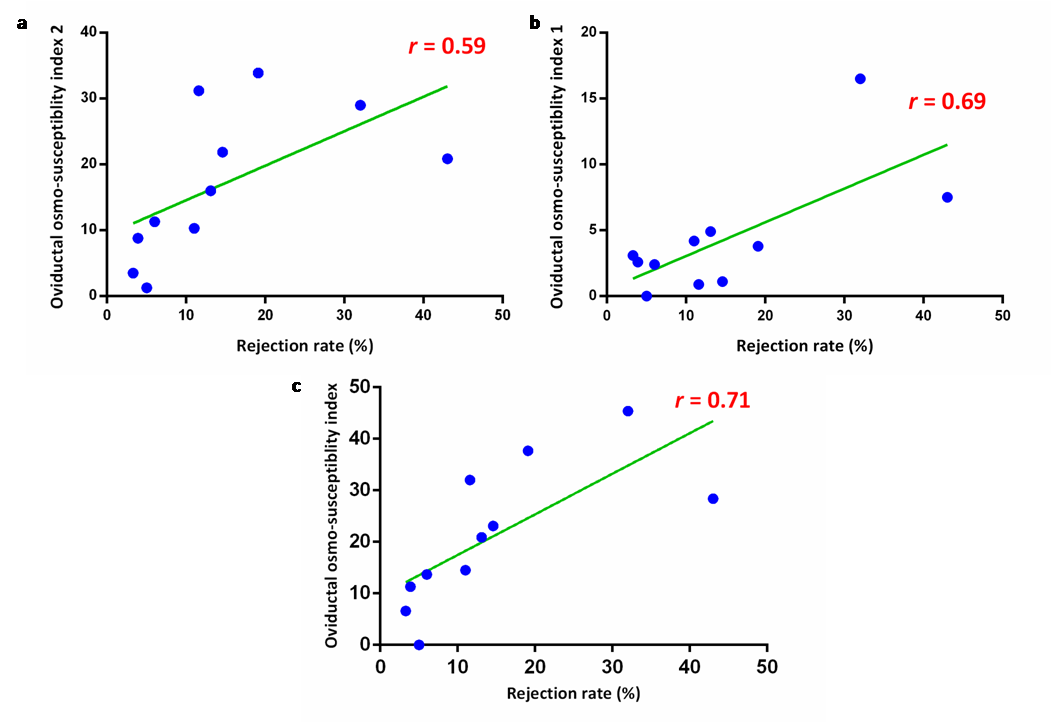


**Supplementary figure S4: Correlation between predicted oviduct osmo-susceptibility indices (oviduct osmo-susceptibility indices at 1 h (OVI 1- a), at 4 h (OVI 4- b) and total score (OVI- c)) and rejection rate. The total osmo-susceptibility index (OVSI) arrived using HYP 1 media with oviduct pH (7.4) was strongly negatively correlated (r = 0.71) with ejaculate rejection rate.**

**Supplementary Tables**

**Supplementary Table S1: The correlation of sperm functional parameters at different incubation media simulating bovine uterus and oviductal osmolality and pH with semen rejection rate.**

| Parameter | **Correlation coefficient (*r*) of sperm functional parameters with rejection rate**  (*p* < 0.05) | | | | | | | | | | | |
| --- | --- | --- | --- | --- | --- | --- | --- | --- | --- | --- | --- | --- |
|  | **ISO 6.8** | | **HYP1 6.8** | | **HYP2 6.8** | | **ISO 7.4** | | **HYP1 7.4** | | **HYP2 7.4** | |
|  | **1 h** | **4 h** | **1 h** | **4 h** | **1 h** | **4 h** | **1 h** | **4 h** | **1 h** | **4 h** | **1 h** | **4 h** |
| **MUP(%)** |  |  |  | -0.80 |  |  |  |  |  |  |  |  |
| **MMP(%)** | -0.60 | -0.57 | -0.67 | -0.60 | -0.50 |  | -0.56 |  | -0.73 | -0.73 | -0.63 |  |
| **FMI (%)** | -0.57 |  | -0.60 | -0.65 | -0.58 | -0.59 | -0.58 | -0.59 | -0.59 | -0.69 | -0.60 |  |
| **AI (%)** |  |  | -0.72 |  | -0.76 |  | -0.72 |  | -0.77 |  | -0.70 |  |

(MUP – mucous penetration, MMP – mitochondrial membrane potential, FMI- functional membrane integrity, AI- acrosome integrity)

**Supplementary Table S2: The correlation of osmo-susceptibility index with rejection rate (%) and sperm functional parameters (p < 0.05)**

| **Parameter** | **DOI** | **Correlation coefficient (r) of osmo-susceptibility index with rejection rate and sperm functional parameters** |
| --- | --- | --- |
| **Rejection rate** |  | **0.71** |
| Progressive motility (%) | 0 h | -0.67 |
|  | 1 h | -0.62 |
|  | 2 h | -0.72 |
|  | 4 h | -0.81 |
| Total motility (%) | 2 h | -0.71 |
|  | 4 h | -0.83 |
| Head area (µm^2^) | 4 h | -0.62 |
| ALH (µm) | 1 h | -0.60 |
|  | 2 h | -0.67 |
|  | 4 h | -0.68 |
| BCF (Hz) | 1 h | -0.67 |
| Mucus penetration (%) | 4 h | -0.59 |
| AR(%) | 1 h | 0.59 |
| MMP (%) | 0 h | -0.75 |
|  | 2 h | -0.74 |
|  | 4 h | -0.92 |
| FMI (%) | 0 h | -0.74 |
|  | 2 h | -0.83 |
|  | 4 h | -0.58 |
| AR(%) | 1 h | 0.59 |

(ALH – amplitude of lateral head displacement, BCF – beat cross frequency, AR – acrosome reacted, MMP – mitochondrial membrane potential, FMI – functional membrane integrity)

**Table S3: The correlation of relative gene expression levels (ΔCt) of osmo-responsive genes with sperm functional parameters**

| **Correlation coefficient (*r*) of relative gene expression levels with sperm functional parameters and rejection rate** (*p* < 0.05) | | | | | | | | |
| --- | --- | --- | --- | --- | --- | --- | --- | --- |
| **Parameter** | ***HSP90AB1*** | ***MT-ND2*** | ***NFAT5*** | ***ADAM1B*** | ***SLC9C1*** | ***MT-CO1*** | ***EFHD1*** | ***ENO1*** |
| **PM (%)** |  |  |  |  |  | -0.48 | -0.46 | -0.46 |
| **Rapid PM (%)** |  |  |  |  |  | -0.54 | -0.63 | -0.62 |
| **VAP (µm/s)** |  |  |  |  |  |  | -0.58 | -0.57 |
| **VSL (µm/s)** |  |  |  |  |  |  | -0.53 | -0.59 |
| **LIN (%)** | -0.47 |  |  |  | -0.47 |  | -0.55 | -0.57 |
| **Hyperactive (%)** |  |  | 0.51 |  |  |  |  |  |
| **Head area (µm^2^)** |  |  | 0.64 | 0.62 |  | -0.45 |  |  |
| **ALH (µm)** | 0.45 |  |  |  |  |  |  |  |
| **BCF (Hz)** |  |  |  |  |  |  | -0.55 |  |
| **Mucus penetration (%)** |  |  |  |  |  | -0.51 | -0.68 | -0.67 |
| **Non capacitated (%)** |  |  | 0.5 | 0.58 | 0.59 |  |  |  |
| **Acrosome intact (%)** |  |  |  | 0.57 | 0.68 |  |  |  |
| **MMP (%)** |  |  | 0.48 | 0.46 |  | -0.55 |  | -0.48 |
| **FMI (%)** |  | -0.49 |  |  |  | -0.51 |  | -0.47 |
| **CD (%)** |  |  | 0.5 |  |  |  | -0.45 |  |
| **RR (%)** | -0.51 |  | -0.74 | -0.71 | -0.49 |  |  |  |

(PM – progressive motility, VAP – average path velocity, VSL – straight-line velocity, LIN- linearity index, ALH – amplitude of lateral head displacement, BCF – beat cross frequency, MMP – mitochondrial membrane potential, FMI – functional membrane integrity, CD – chromatin distribution, RR – rejection rate)

**Supplementary Table S4: The validation of oviductal osmo-susceptibility index cut-off in randomly chosen semen samples. The chosen 18% cut-off segregated the samples effectively.**

| **Animal ID** | **Osmo-susceptibility index value** | **Ejaculate rejection rate** | **Quality of the bull** |
| --- | --- | --- | --- |
| **1** | 8.51 | 3.3 | Good semen producer |
| **2** | 17.81 | 5 |  |
| **3** | 2.63 | 9.1 |  |
| **4** | 21.02 | 11 | Poor semen producer |
| **5** | 38.99 | 15.7 |  |
| **6** | 50.32 | 21.6 |  |

**Methodology :**

**Supplementary data I**

**Fixing of sampling time points for osmo-adaptation study**

The osmotic stress is expected to cause perturbation of cell volume and thus may transiently affect the motility of sperm. Since the window of this osmo-adaptive behavior in sperm was not studied before, there was a necessity to conduct a pilot study to fix the sampling time points after subjecting to osmotic stress. The sperm was subjected to hyperosmotic stress (HYP1: 355mOsm/kg; HYP2: 420 mOsm/kg) with a control (ISO: 290 mOsm/kg). An aliquot of semen sample was taken immediately after incubation (0 h) and in equal intervals of 30 min (0.5, 1.0, 1.5, 2.0, 2.5, 3.0, 3.5, 4.0 h) and subjected to motility analysis and HOS test.

1. **Progressive forward motility**

The progressive motility (%) in the hyperosmotic media reduced significantly (*p*< 0.05) on immediate (0 h) exposure to stress and subsequently regained over a period of 1- 2 h (Supplementary data table 1), whereas continuous drop was observed in the isosmotic medium. The time point at which maximum motility attained after a initial drop was considered as the regaining ability of that sample. Further, the time taken for regaining the motility varied from 1-2 h among the bulls. The regained progressive motility in the hyperosmotic medium (HYP1) significantly sustained well up to 4 h as compared to isosmotic medium (Supplementary table T5).

**Supplementary data table 1:** Standardization of duration of incubation for the experiment based on the changes in progressive motility (%) of sperm subjected to different osmotic media (n=6) (DOI: Duration of incubation; ISO: 290 mOsm/kg; HYP1: 355 mOsm/kg; HYP2: 420 mOsm/kg; BI: Before incubation)

| **DOI**  **(h)** | **Progressive motility (%)** | | |
| --- | --- | --- | --- |
|  | **ISO 6.8** | **HYP1** | **HYP 2** |
| BI | 88.1±1.33 | 88.1±1.33^X^ | 88.1±1.33^X^ |
| 0.0 | 85.5±1.71 | 65.3±3.27^Y^ | 56.1±6.71^Y^ |
| 0.5 | 78.0±4.31 | 76.9±3.78^Z^ | 73.4±5.52^Z^ |
| 1.0 | 76.1±3.51 | 77.7±3.65 | 77.2±4.85 |
| 1.5 | 73.5±3.28 | 77.2±2.91 | 74.3±4.13 |
| 2.0 | 71.6±3.91 | 75.0±2.84 | 76.2±3.83 |
| 2.5 | 65.5±5.14 | 74.5±2.75 | 69.1±5.50 |
| 3.0 | 63.5±4.71 | 72.2±4.17 | 63.5±4.78 |
| 3.5 | 58.4±3.67 | 66.4±4.68 | 60.1±5.48 |
| 4.0 | 54.6±3.69^a^ | 64.5±5.18^b^ | 56.0±6.48^a^ |

Superscript bearing ^a,b^ in a row differ significantly (*p*<0.05)

Superscript bearing ^X,Y,Z^ in a column differ significantly (*p*<0.05)

1. **Functional membrane integrity**

The osmotic stress did not have adverse effect on percentage of sperm functional membrane integrity. Instead, there was a non-significant conservative effect of functional membrane integrity in the hyperosmotic media especially in HYP1 as evidenced from 3 h of incubation (Supplementary data table 2). The percentage of sperm progressive motility decreased immediately at 0 h and regained between 1-2 h, whereas the percentage of functional membrane integrity sustained in hyperosmotic media from 3 h. Based on these preliminary observation, it was decided to retrieve samples at 0, 1, 2 and 4 h for further studies.

**Supplementary data table 2:** Standardization of duration of incubation for the experiment based on the changes in functional membrane integrity (%) of sperm subjected to hyperosmotic media(n=6).(DOI: Duration of incubation; ISO: 290 mOsm/kg; HYP1: 355 mOsm/kg; HYP2: 420 mOsm/kg; BI:Before incubation)

| **DOI**  **(h)** | **Functional membrane integrity (%)** | | |
| --- | --- | --- | --- |
|  | **ISO 6.8** | **HYP1** | **HYP 2** |
| **BI** | 84.0±1.34 | 84.0±1.34 | 84.0±1.34 |
| **0.0** | 82.3±1.17^a^ | 80.2±1.62^ab^ | 78.5±1.23^b^ |
| **0.5** | 79.3±2.33^a^ | 75.5±2.05^ab^ | 74.8±1.64^b^ |
| **1.0** | 70.8±1.49 | 70.2±1.64 | 68.3±2.03 |
| **1.5** | 68.5±1.65 | 65.5±2.72 | 62.8±2.95 |
| **2.0** | 63.2±3.19 | 62.2±2.89 | 59.7±3.29 |
| **2.5** | 56.0±4.60 | 57.0±3.37 | 58.2±4.43 |
| **3.0** | 48.3±4.88 | 52.0±3.76 | 52.5±4.30 |
| **3.5** | 43.0±4.84 | 46.0±3.07 | 47.7±3.83 |
| **4.0** | 37.5±4.98 | 42.2±2.50 | 41.7±3.33 |

Superscript bearing ^a,b^ in a row differ significantly (*p*<0.05)
