# Supplementary figures and images for: Sperm preparedness and adaptation to osmotic and pH stressors relate to functional competence of sperm in Bos taurus
Source: Sci Rep. 2021 Nov 19;11:22563. doi: 10.1038/s41598-021-01928-6 (PMC8604908; doi:10.1038/s41598-021-01928-6)

**a**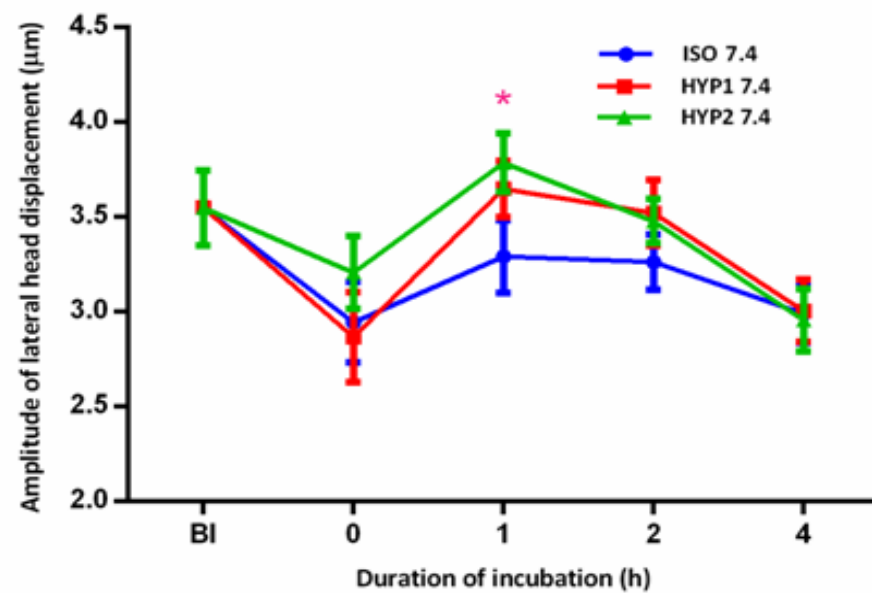**b**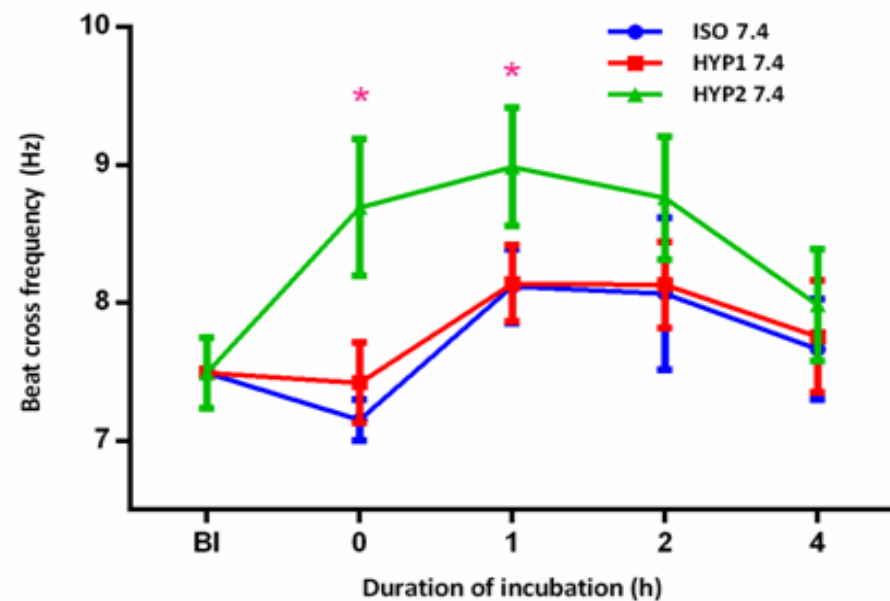

Supplement: Supplementary file 2 — Supplementary Information 2. [file 41598_2021_1928_MOESM2_ESM.pdf]

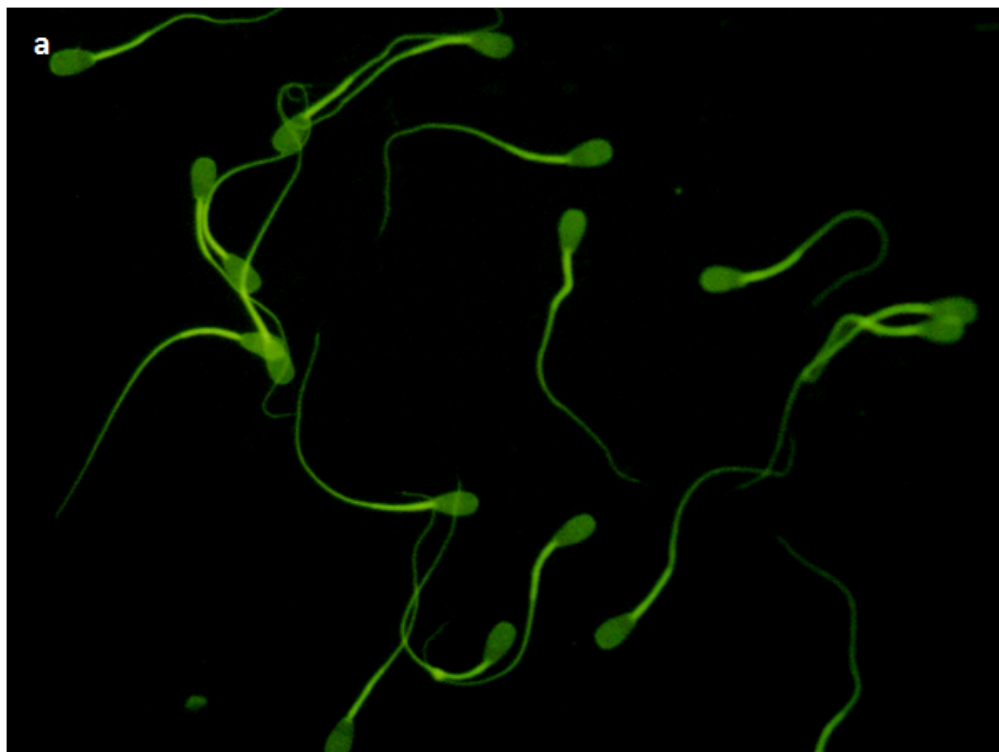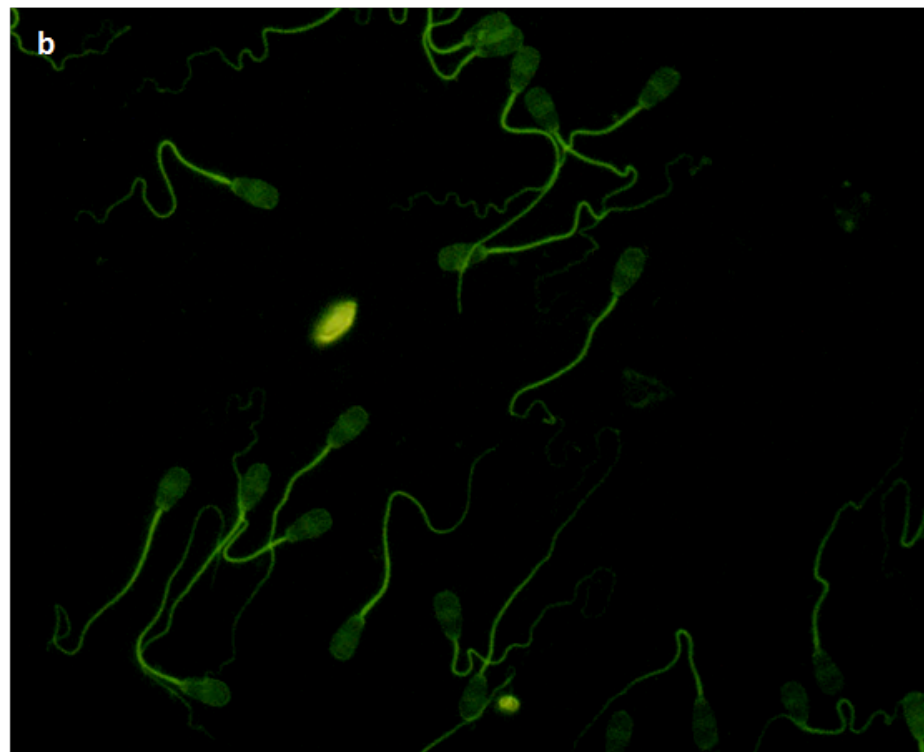

Supplement: Supplementary file 3 — Supplementary Information 3. [file 41598_2021_1928_MOESM3_ESM.pdf]

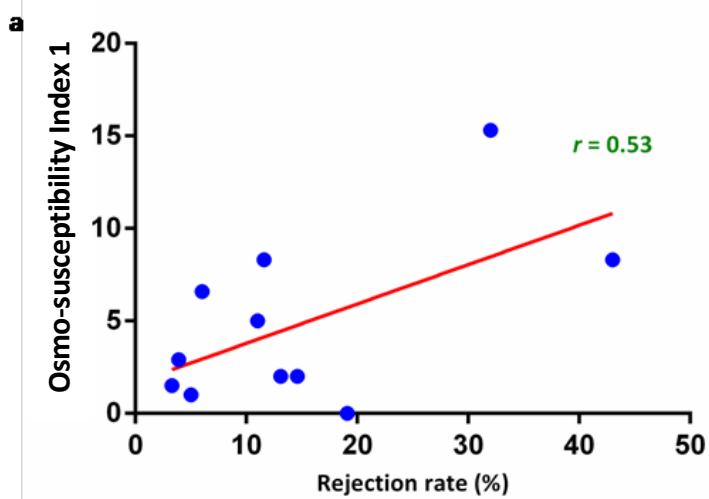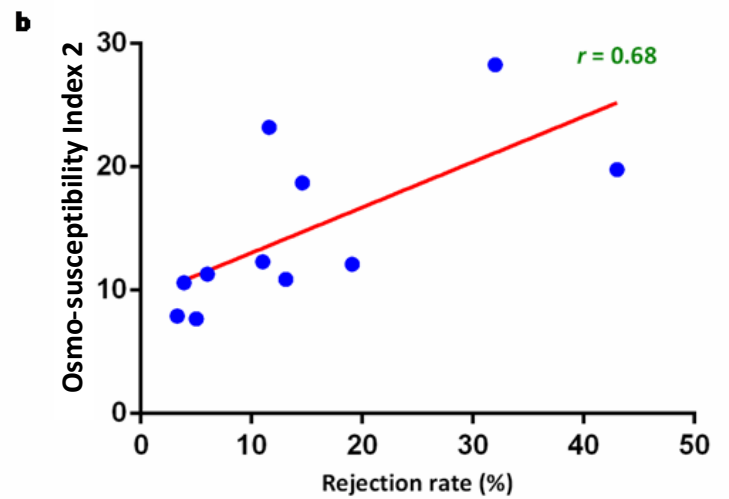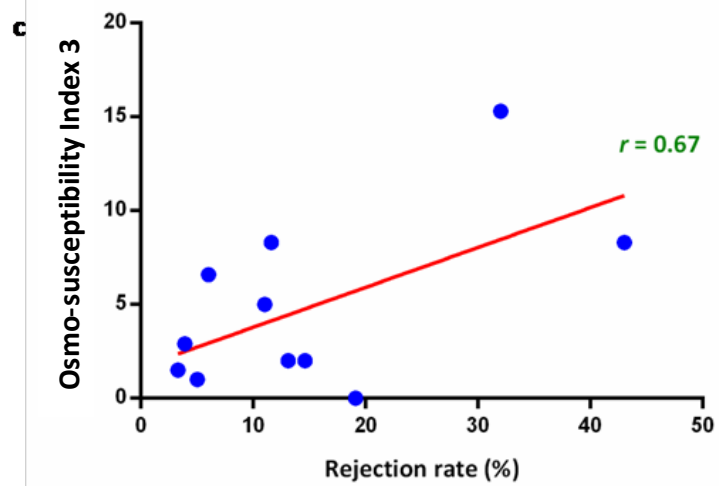

Supplement: Supplementary file 4 — Supplementary Information 4. [file 41598_2021_1928_MOESM4_ESM.pdf]

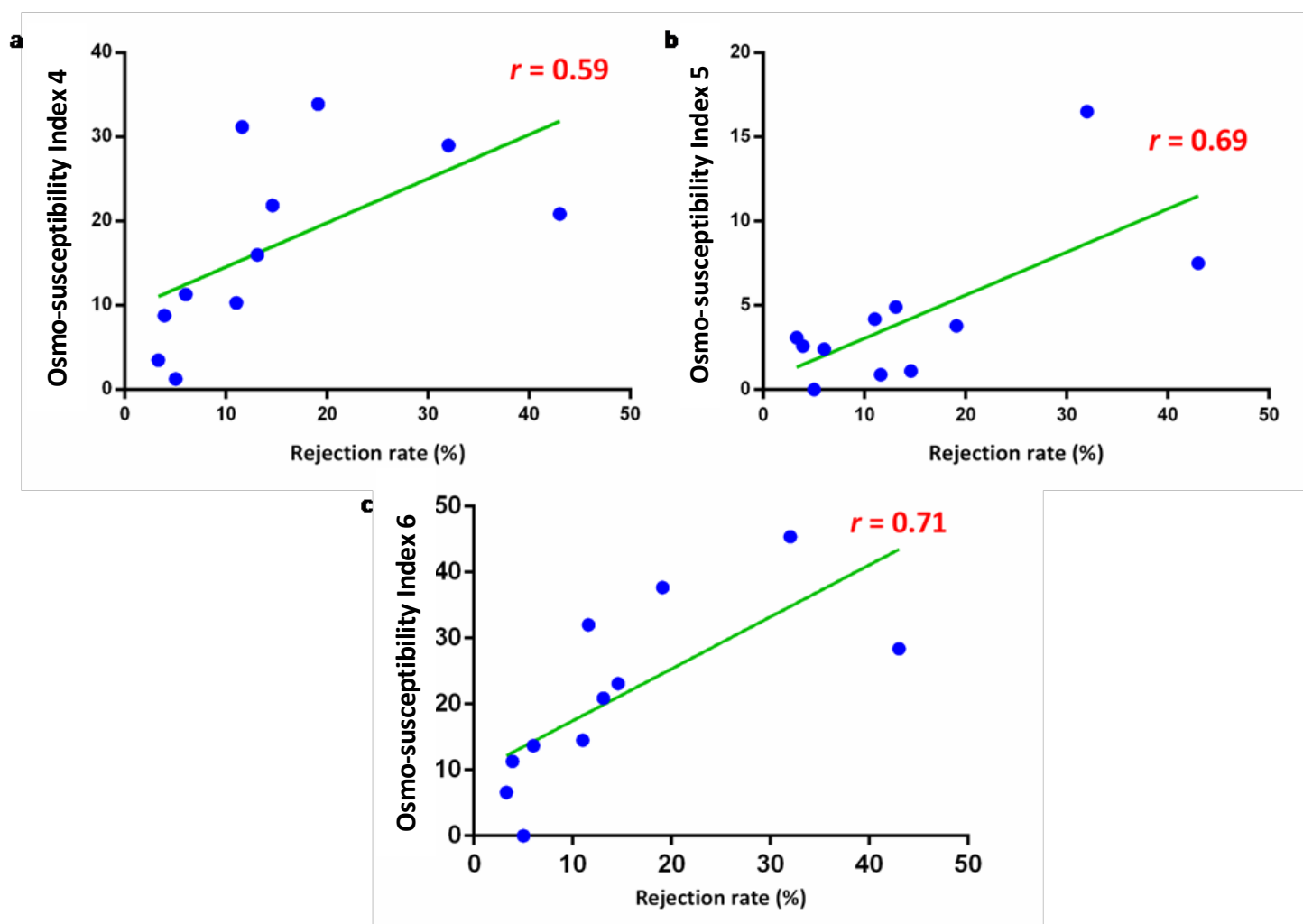

Supplement: Supplementary file 5 — Supplementary Information 5. [file 41598_2021_1928_MOESM5_ESM.pdf]
